# A case report of an atypical presentation of pyogenic iliopsoas abscess

**DOI:** 10.1186/s12879-019-3675-2

**Published:** 2019-01-17

**Authors:** Bang Yu Xu, Farhad Fakhrudin Vasanwala, Sher Guan Low

**Affiliations:** 1 Department of Family Medicine, Sengkang Health, SingHealth, 110 Sengkang East Way, Sengkang, 544886 Singapore; 2 Department of Post Acute and Continuity Care, Sengkang Community Hospital, 1 Anchorvale St, Sengkang, 54483 Singapore

**Keywords:** Case report, Atypical presentation, Iliopsoas abscess

## Abstract

**Background:**

Iliopsoas abscess is a collection of pus in the iliopsoas muscle compartment. It can be primary or secondary in origin. Primary iliopsoas abscess occurs as a result of hematogenous or lymphatic seeding from a distant site. This is commonly associated with a chronic immunocompromised state and tends to occur in children and young adults. Secondary iliopsoas abscess occurs as a result of the direct spread of infection to the psoas muscle from an adjacent structure, and this may be associated with trauma and instrumentation in the inguinal region, lumbar spine, or hip region. The incidence of iliopsoas abscess is rare and often the diagnosis is delayed because of non-specific presenting symptoms.

**Case presentation:**

We describe a patient with iliopsoas abscess who presented to the Emergency Department at X Hospital on three separate occasions with non-specific symptoms of thigh pain and fever before finally being admitted for treatment. This case illustrates how the diagnosis can be delayed due to its atypical presentation. Hence, highlighting the need for clinicians to have a high index of clinical suspicion for iliopsoas abscess in patients presenting with thigh pain and fever.

**Conclusion:**

The classic triad of fever, flank pain, and hip movement limitation is presented in only 30% of patients with iliopsoas abscess. Clinicians should consider iliopsoas abscess as a differential diagnosis in patients presenting with fever and thigh pain. The rare condition with the varied clinical presentation means that cross-sectional imaging should be considered early to reduce the risk of fulminant sepsis.

## Background

Iliopsoas abscess is a collection of pus in the iliopsoas muscle compartment. The psoas muscle arises from the tip of the transverse processes and the lateral aspects of the vertebral bodies between the 12th thoracic and the 5th lumbar vertebrae. It courses inferiorly across the pelvic brim, anterior to the capsule of the hip joint and beneath the inguinal ligament, forming a tendon with the iliacus muscle and eventually inserts into the lesser trochanter of the femur. The iliacus and psoas muscles are the main hip flexors.

Iliopsoas abscess can be primary or secondary in origin. Primary iliopsoas abscess arises from a distant site of infection which spreads via the haematogenous or lymphatic drainage system. This is commonly associated with a chronic immunocompromised state such as Diabetes Mellitus, Chronic Renal Failure or AIDS. It tends to occur in children and young adults. The direct spread of infection to the psoas muscle from an adjacent structure results in secondary iliopsoas abscess. This may be associated with trauma and instrumentation in the lumbar spine, inguinal region or hip region.

Iliopsoas abscess is a rare medical condition. Bartolo DC et al. [1] reported an incidence of iliopsoas abscess of 0.4/100000 in the United Kingdom. Iliopsoas abscess tends to affect more male patients, with a male: female ratio of 1.62:1 as reported by Lai YC et al. [2]. The reported mortality of iliopsoas abscess is up to 19% [3]. The symptoms of iliopsoas abscess [4] include fever, vague flank pain, loss of appetite and weight, lump in the inguinal region and/or limited range of movement of the hip joint. Because of non-specific presenting symptoms, the diagnosis of iliopsoas abscess is often delayed. The classic triad of fever, flank pain, and limited range of movement of the hip joint is present in only 30% of patients with iliopsoas abscess [5]. *Staphylococcus aureus* is the most common causative organism in primary iliopsoas abscess while *Escherichia coli* is main causative organism in secondary iliopsoas abscess [2]. Management of iliopsoas abscess depends on the both patient and disease factors. Patient factors include pre-existing medical conditions and fitness for surgery. Disease factors include size of iliopsoas abscess and causative organisms. Treatment of iliopsoas abscess consists of drainage and prompt initiation of appropriate antibiotic [4]. Most small abscesses may be treated with antibiotics only while majority of those that require drainage can be done effectively with imaging modalities such as Ultrasound-guided or Computerized Tomography-guided drainage [6]. Antibiotic therapy should also be tailored to the causative organisms isolated.

We describe a patient with iliopsoas abscess who presented to the Emergency Department at X Hospital on three separate occasions, and was eventually admitted for definitive treatment. This illustrates how the diagnosis can be delayed due to its atypical presentation.

## Cases presentation

Mr. A was a 38 year old gentleman from China. He worked as a cleaner in Singapore for the past three years. He was ADL-independent and community ambulant and denied any pre-existing medical conditions prior to this illness. He did not smoke or consume any alcohol. He first presented to X hospital Emergency Department with a history of left thigh pain for seven days. The pain was sharp with a pain score of 6/10, radiating down to his left leg. He had difficulty walking as well. He felt feverish but had no other localizing symptoms to suggest infection. Mr. A denied any recent travel history.

On examination, he was afebrile and his blood pressure was 120/75 mmHg with a pulse rate of 90/min. His gait was slightly antalgic, worse on the left lower limb. On examination of his left thigh, there was no swelling or erythema. Tenderness was elicited on palpation of his upper inner thigh area. Examination of his left hip demonstrated a slight limitation in ROM due to pain. Examination of his left knee was unremarkable. His left lower limb distal pulses were well felt. No portal of wound entry was found on examination of his lower limbs.

His blood tests showed raised inflammatory markers with a white cell count of 10.4 × 10^9^/L but his neutrophil count was normal at 45%. His urea and electrolytes were normal and X-ray of his left hip was unremarkable. The impression was musculoskeletal pain, and he was discharged with analgesia. C-reactive protein was not done as the clinical suspicion of infection was low by the attending emergency physician.

He attended the Emergency Department three days later with the same complaint. His examination findings were unremarkable as recorded by the attending emergency physician. X-rays of his femur and tibia/fibula were unremarkable and he was discharged with analgesia.

He attended the Emergency Department for the third time seven days later as he developed a painful lump over the medial aspect of his left upper thigh. On examination he was febrile with temperature 38.4 °C and his blood pressure was 114/75 mmHg with a pulse rate of 96/min. A 6 cm × 7 cm area of erythematous induration with pus-discharging sinus was seen over the medial border of his left upper thigh. No soft tissue crepitus was felt during palpation. The range of movement of his left hip was limited due to pain. The distal pulses of his left limb were felt and both his calves were supple.

**Fig. 1:**
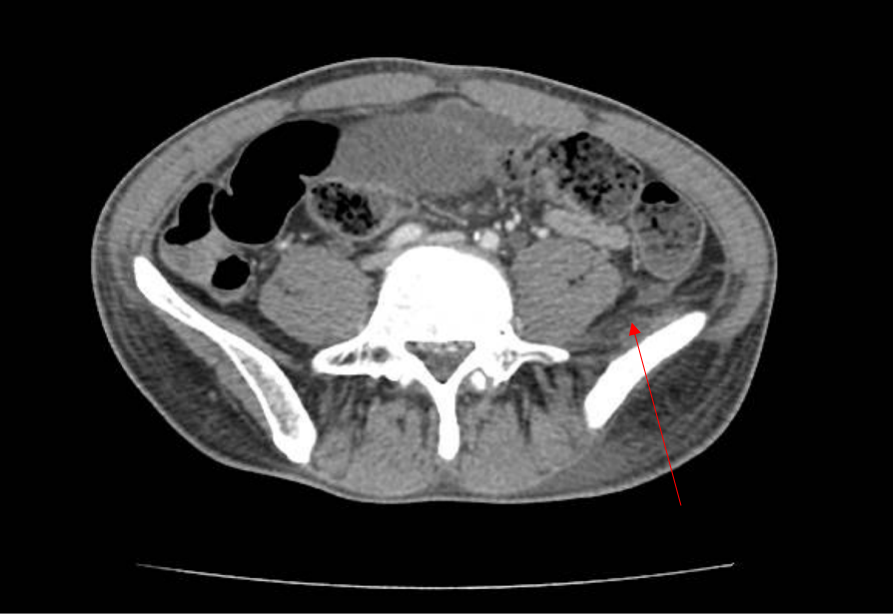
CT Abdomen and Pelvis cut demonstrating an irregular rim-enhancing fluid collection centred over the left medial thigh, involving the left abductor muscles, extending superiorly to involve the obturator externus and iliacus muscles to the level of the iliac crest

**Fig. 2:**
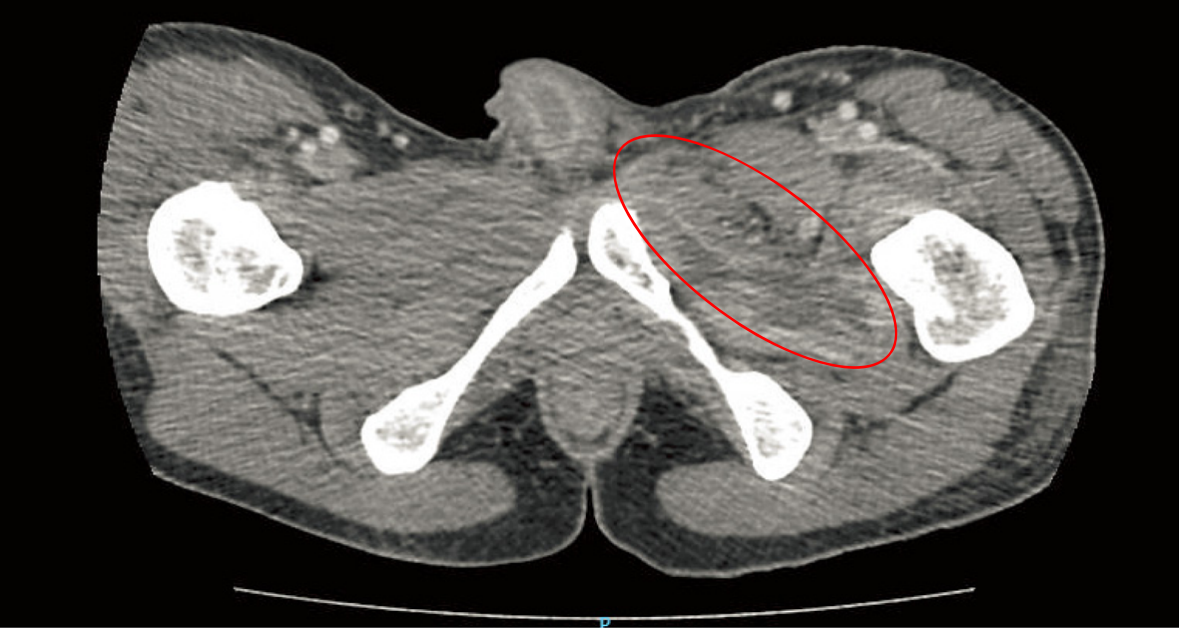
CT Abdomen and Pelvis cut demonstrating the collection inferiorly extends to a soft tissue defect over the medial thigh, with the largest pocket at the adductor muscles measures approximately 8.8 × 2.7 cm

His blood tests showed significantly raised inflammatory markers with a white cell count of 14.8 × 10^9^/L with a raised neutrophil count at 83% and C-reactive protein of 305 mg/L. His urea and electrolytes were normal. CT of his Abdomen and Pelvis demonstrated an irregular rim-enhancing fluid collection centred over the left medial thigh, involving the left abductor muscles, extending superiorly to involve the obturator externus and iliacus muscles to the level of the iliac crest (Fig. 1). Inferiorly, the collection extends to a soft tissue defect over the medial thigh. The largest pocket at the adductor muscles measures approximately 8.8 × 2.7 cm (Fig. 2). There is swelling of the involved muscles, including the left psoas with mild fat stranding and thickening of the adjacent peritoneum over the left iliac fossa.

He was admitted for incision and drainage underwent the procedure under General Anaesthesia. Empirical intravenous cefazolin was initiated. His intra-operative finding was that of a deep-seated adductor compartment abscess with a copious amount of pus, extending to the left iliacus region. Both his blood and wound cultures grew MSSA. His Transesophageal Echocardiography excluded infective endocarditis. His fasting glucose, HIV and hepatitis screening were negative. His intravenous antibiotic regime was switched to six weeks of cloxacillin, and a PICC was inserted.

He underwent three wound debridement surgeries. During routine wound inspection after first two surgeries, pus can still be expressed from the wound edges and wound bed appears unhealthy. This could likely be due to the tracking down of the pus from the involved the obturator externus and iliacus muscles after the first incision and drainage of the thigh abscess. Eventually his thigh wound healed by secondary intention. He completed his six week course of cloxacillin, and his inflammatory markers came steadily down. A repeat CT three months later demonstrated interval resolution of the previously seen left iliopsoas and adductor abscess collection.

## Discussion

This case demonstrates that the presentation of a psoas abscess continues to be a diagnostic challenge for clinicians. Mr. A had presented to the Emergency department with non-specific symptoms of fever and left thigh pain. This acute presentation which was not related to trauma could suggest an underlying inflammatory process. A thorough clinical history should be taken to distinguish between pain that is inflammatory or mechanical in nature. Mechanical pain typically has an acute onset, worsened with physical activity or joint movement, relieved with rest and not associated with morning stiffness. Inflammatory pain often has insidious onset, worsened with rest, relieved with physical activity and often associated with early morning stiffness. The level of suspicious for an underlying inflammatory process should be raised if associated with significant family history of chronic inflammatory diseases. Inflammatory markers such as Erythrocyte Sedimentation Rate (ESR) and C-reactive protein (CRP) should be obtained in patient who had fever associated with muscle pain. His initial atypical presenting features of thigh pain and fever should raise suspicion, although the classical clinical triad of flank pain, fever, and limitation of the hip movement were absent. Al Shehri DM et al. [7] reported a case of late onset iliopsoas abscess due to stump appendicitis. Cargill T et al. [8] reported a case of pyogenic iliopsoas abscess with an uncommon presentation of non-specific leg pain. The varied clinical presentation of iliopsoas abscess made the diagnosis challenging.

Limitation of hip movement is common in patients with iliopsoas abscess and patients frequently preferred to be in a position of hip flexion and lumbar lordosis. Pain is exacerbated when performing movements in which the psoas muscle is stretched. This “psoas sign” is pain brought on by extension of the hip. The “psoas sign” together with diminished hip pain during hip flexion may be useful in providing a clue for the clinician to diagnose the condition.

MRI of the thigh may be useful for early diagnosis of muscle abscess. The MRI characteristics of lesions producing a mass effect are variable and typically different from those of normal muscle on all pulse sequences. Muscle abscess may be associated with an intramuscular mass lesion and MRI provides a characterization of mass composition that allow clinicians to diagnose muscle abscess early [9]. For instance, a T2 weighted MR image demonstrating diffuse oedema with intramuscular gas indicates the presence of gas gangrene.

Mr. A is likely to have secondary iliopsoas abscess, possibly arising from an insidious injury to his upper left thigh which resulted in a thigh abscess with direct spread of infection to the iliopsoas muscle.

## Conclusion

Iliopsoas abscess should be included in the differential diagnoses in patients presenting with fever, leg pain, antalgic gait with limited hip movement. Educating clinicians on the varied clinical presentation of iliopsoas abscess is important in order to improve recognition of this condition. Early imaging should be considered to reduce the risk of complications such as septic shock and to improve outcomes.

## Abbreviations

AIDS: Acquired Immunodeficiency Syndrome
CT: Computerized Tomography
HIV: Human Immunodeficiency Virus
MRI: Magnetic Resonance Imaging
MSSA: Methicillin-Sensitive *Staphylococcus aureus*
PICC: Peripherally-Inserted Central Catheter
ROM: Range of Motion
